# Acquisition of basic ear surgery skills: a randomized comparison between endoscopic and microscopic techniques

**DOI:** 10.1186/s12909-019-1803-8

**Published:** 2019-09-14

**Authors:** Lukas Anschuetz, Daniel Stricker, Abraam Yacoub, Wilhelm Wimmer, Marco Caversaccio, Sören Huwendiek

**Affiliations:** 1 Department of Otorhinolaryngology, Head and Neck Surgery, Inselspital, University Hospital and University of Bern, Freiburgstrasse, CH-3010 Bern, Switzerland; 2 Institute for Medical Education, Department for Assessment and Evaluation, University of Bern, Bern, Switzerland; 3 ARTORG Center for Biomedical Engineering, Hearing Research Laboratory, University of Bern, Bern, Switzerland

**Keywords:** Surgical skills, Education, Teaching, Endoscopic ear surgery, Microscope, Endoscope

## Abstract

**Background:**

Endoscopic ear surgery is gaining increasing popularity and has an important impact on teaching middle ear anatomy and basic surgical skills among residents and fellows. Due to the wide-angled views offered, the approach significantly differs from the established microscopic technique. This randomized study compares the acquisition of basic ear-surgery skills using the endoscopic and microscopic technique under standardized conditions. We aim to investigate the required surgical times, attempts and accidental damages to surrounding structures (errors) in surgeons with different training levels.

**Methods:**

Final-year medical students (*n* = 9), residents (*n* = 14) and consultants (*n* = 10) from the Department of Otorhinolaryngology, Head and Neck Surgery at the University Hospital of Bern, Switzerland were enrolled in the present study. After randomization every participant had to complete a standard set of grasping and dissecting surgical tasks in a temporal bone model. After the first session the participants were crossed over to the other technique.

**Results:**

Time required for completion of the surgical tasks was similar for both techniques, but highly dependent on the training status. A significant increase in the number of damages to the ossicular chain was observed with the microscopic as compared to the endoscopic technique (*p* < 0.001). Moreover, students beginning with the endoscopic technique showed an overall significantly lower amount of time to complete the tasks (*p* = 0.04). From the subjective feedback a preference towards the endoscopic technique mainly in medical students was observed.

**Conclusions:**

The endoscopic approach is useful and beneficial for teaching basic surgical skills, mainly by providing a reduction of damage to surrounding tissues with similar operating times for both techniques. Moreover, medical students performed significantly faster, when first taught in the endoscopic technique. Especially for young surgeons without previous training in ear surgery, the endoscope should be considered to improve surgical skills in the middle ear.

## Background

Endoscopic ear surgery (EES) is an emerging technique to treat pathologies of the middle ear and the lateral skull base. The use of rod lens endoscopes inside the middle ear provides the surgeon with wide-angled panoramic views of the anatomy and pathology. Moreover, angled scopes reach hidden areas of the middle ear such as the retro- and hypotympanum [1–3]. These technical refinements allow the surgeon to treat middle-ear pathologies adopting a minimally invasive transcanal approach without skin incision or removal of bone for access purposes. The clinical efficacy of the endoscopic technique compared to the standard microscopic technique has previously been shown in various fields of middle ear surgery like type I tympanoplasties [4], cholesteatoma [5] and stapes surgery, especially in stapes-malformation cases [6].

Despite the advantages mentioned above, exclusive EES has its limitations and technical particularities. The most important differences regarding the acquisition of surgical skills compared to the microscopic approach are probably the one-handed surgical technique and the two-dimensional view. Since one hand is holding and guiding the endoscope, only one hand remains to perform the appropriate surgical steps. As a result, the surgical learning curve is deemed to be flatter, requiring more training to achieve a certain level compared to the microscopic technique.

Regarding training, a wide variety of simulators have been reported in the literature for surgical training in otolaryngology [7]. In otology training, Musbahi et al. [8] identified 32 different surgical simulators. Most simulators and investigations focus on mastoidectomy training using a microscopic technique. More recently, simulators for exclusive EES have been developed and validated, with different modalities such as 3D-printed models [9], custom build simulators [10], cadaveric dissection programs [11] and animal models [12]. A recently published validation showed statistically significant improvement of surgical skills in medical students, when using an EES simulator [13].

However, no comparative studies between these techniques have been performed to the best of our knowledge. Therefore, a dedicated investigation regarding skills acquisition with the above mentioned two methods in the setting of middle ear surgery is required. We hypothesize, that especially young surgeons, who have not been previously trained in middle-ear surgery may have different outcomes compared to consultants, since their mental models of surgery have not been developed yet.

This randomized study compares the acquisition of basic ear-surgery skills using the endoscopic and microscopic technique under standardized laboratory conditions. We aim to investigate the required surgical times, attempts and accidental damages to surrounding structures (errors) in surgeons with different training levels. Having answers to these questions might have implications on how to acquire and teach ear-surgery skills.

## Methods

This study was reviewed and approved by the local ethical committee (KEK-BE ID REQ-2018-00310), which granted exemption from formal ethical approval for this type of study.

### Participants

**Fig. 1:**
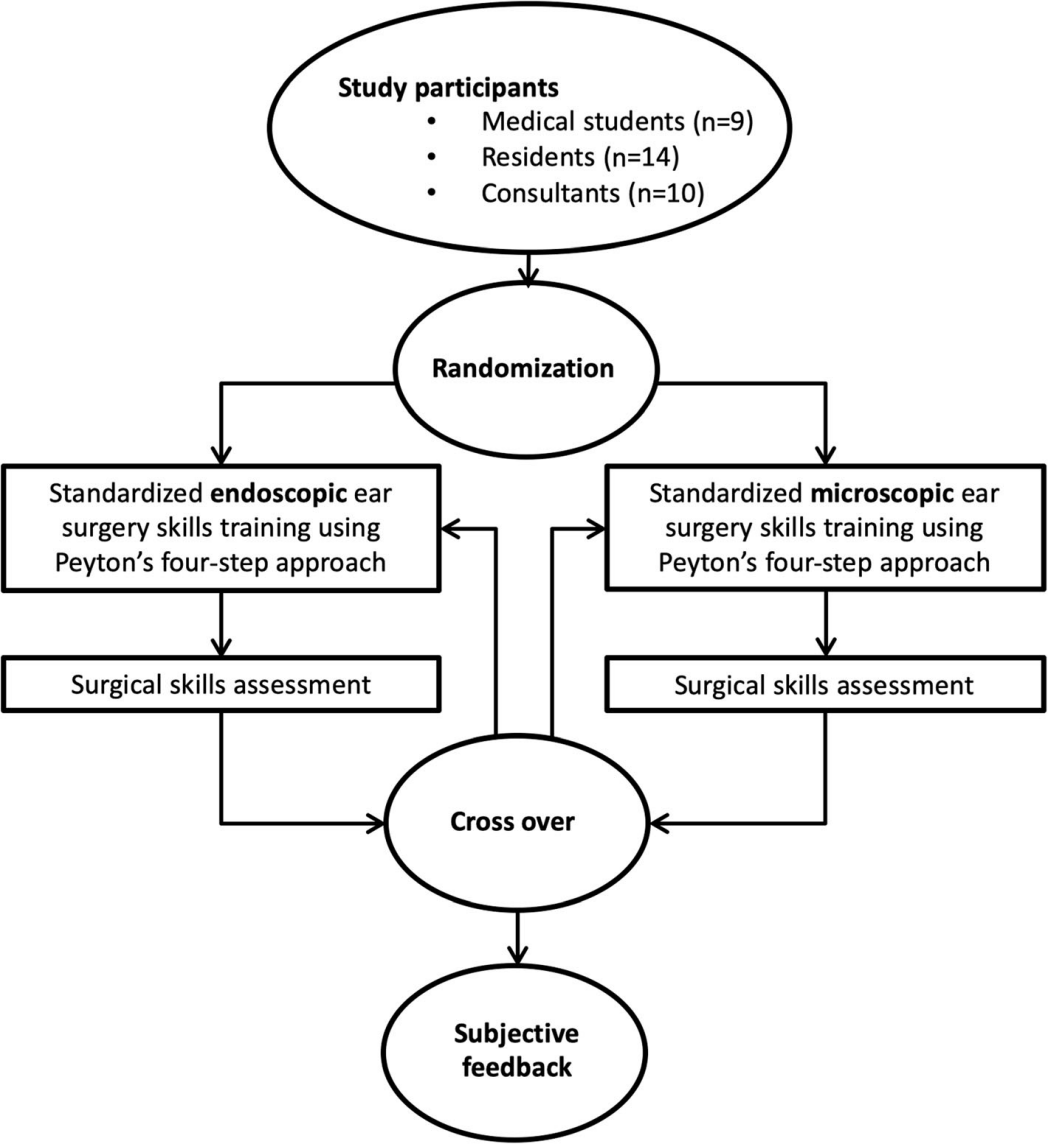
Study design. Flowchart of the different study interventions and assessments

To answer the study questions, we invited final-year medical students, residents and consultants from the Department of Otorhinolaryngology, Head and Neck Surgery (ORL-HNS) at the University Hospital of Bern, Switzerland to participate in the present study. The participants enrolled gave their written consent to participate and were thereafter randomized into two groups containing the same proportion of students, residents and consultants. Group 1 started with the endoscopic technique, whereas group 2 started with the microscope. Afterwards, the groups were crossed-over to the other technique respectively. The study design is summarized in Fig. 1.

### Preparation of cadaveric model

A right, Thiel-fixed, earblock specimen (temporal bone with intact external auditory canal and pinna) was used as model for the various dissection tasks. The experiments were carried out using a rod lens endoscope of 3 mm diameter and 14 cm length connected to a high-definition camera system (Karl Storz, Tuttlingen, Germany) and a surgical microscope (Leica Microsystems, Wetzlar, Germany) used with a 7.5 mm ear speculum rigidly fixed to the surgical Table. A basic set of otologic instruments was available (suction, needle, 0.3 mm hook, micro-forceps, round knife).

**Fig. 2:**
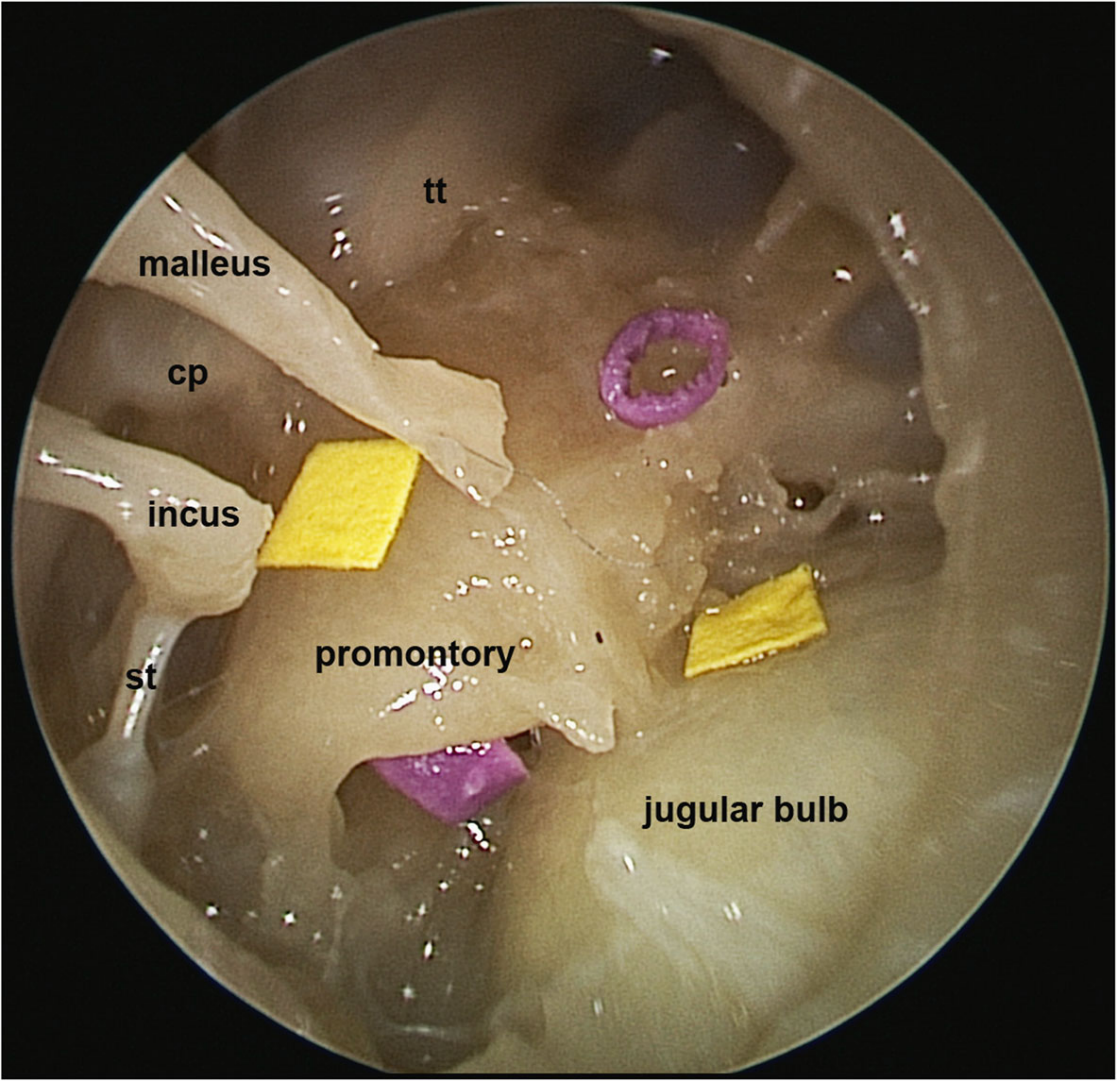
Surgical task setup. The dissection task is simulated by adhesive stickers (yellow), positioned near the cochleariform process and on the jugular bulb. The grasping exercises are simulated by two small plastic rings (violet) positioned in the round window niche and in the protympanic space. The position of the foreign bodies was chosen for equal visibility and accessibility for both techniques. *st: stapedial tendon; cp: cochleariform process; tt:tensor tympani*

The tympanomeatal flap was elevated and positioned in the anterior part of the external auditory canal. To simulate a dissection task, we used adhesive stickers, which were positioned near the cochleariform process and on the jugular bulb. The grasping exercises were simulated by using two small plastic rings positioned in the round window niche and in the protympanic space. The position of the foreign bodies was predefined, standardized and chosen for equal visibility and accessibility for both techniques (Fig. 2).

### Basic surgical-skills acquisition

The instructions for basic surgical-skills acquisition were standardized as indicated below, independently of the technique used (endoscope or microscope) or the educational level of the participants and repeated before each session. Moreover, to minimize differences in instructions given, all dissection sessions were led by the same tutor (LA).

The basic surgical skills were taught according to Peyton’s four-step approach [14]. First the tutor showed the dissection skills to the participants in small groups of three or four in usual speed, followed by a slow step-by-step demonstration with explanations regarding the surgical technique and appropriate instrument handling. Thirdly, the participants were asked to instruct the tutor as how to complete the tasks before they finally performed the dissection and grasping tasks themselves.

The assessment of participant’s performance included: (1) time required to fulfil each task separately; (2) attempts required per task, defined as removal of all instruments before continuing dissection; (3) damages to the ossicular chain per task defined as involuntary contacts to the ossicles and (4) salvage procedures required by the tutor if the participant lost the assigned foreign body in the middle ear and was not able to remove it autonomously.

After 3 to 4 weeks the participant groups switched technique for a second dissection session. Which means that the group initially allocated to the endoscopic technique performed the same educational program using the microscope and the microscopic group was trained to use the endoscope.

### Learners’ perception

**Fig. 3:**
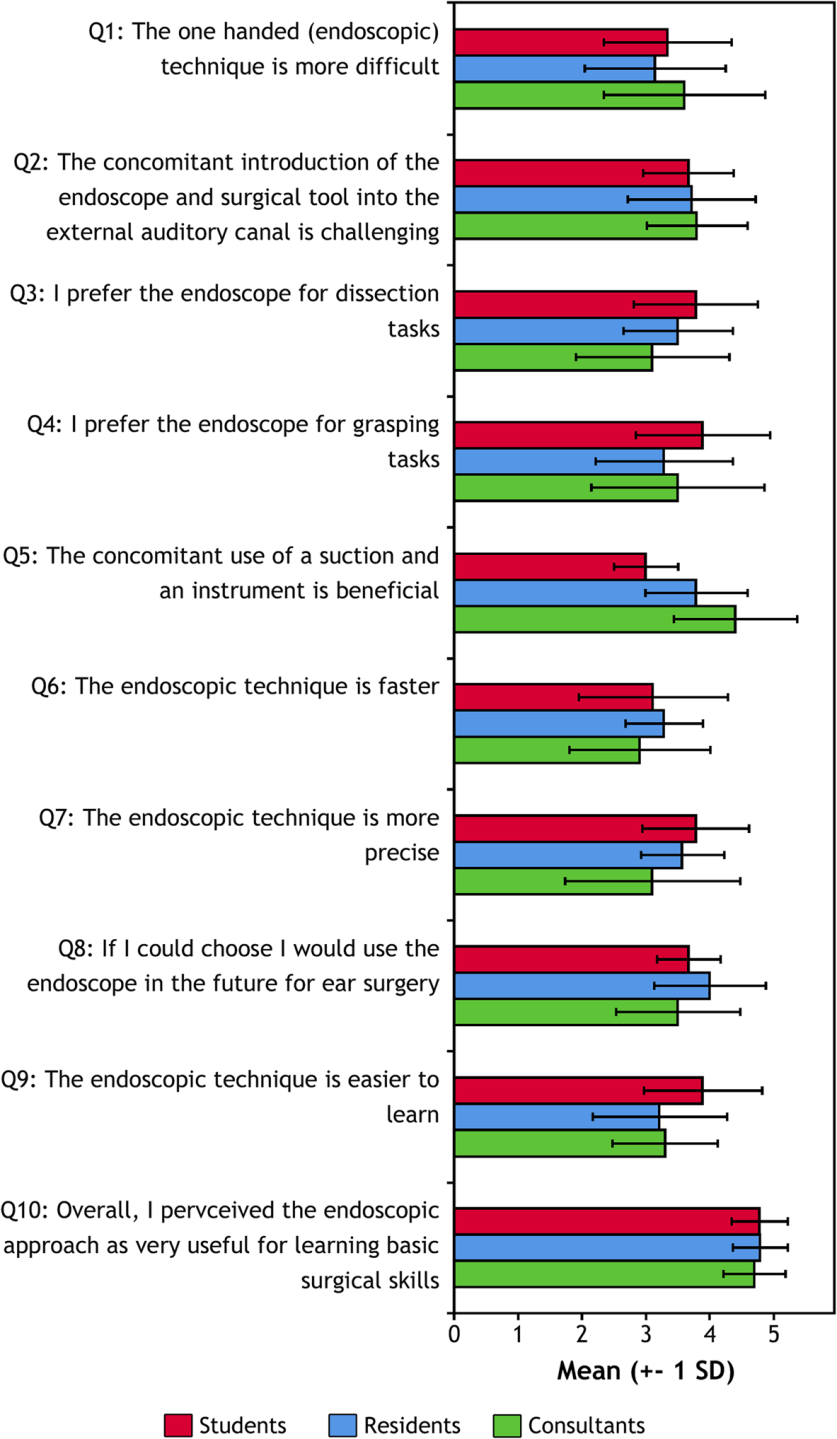
Learner’s perception. At the end of both dissection sections the participants answered ten questions on a 5-point Likert scale comparing both techniques. Note the differences between novice and experiences surgeons

At the end of session 2, subjective feedback was collected using a questionnaire with a five-point Likert scale as answering format. Before the study a think aloud process with 6 participants was done to ensure the intended understanding of the questions. The questions on the questionnaire are shown in Fig. 3.

### Statistical analyses

All data were exported to the Statistical Package for Social Sciences (SPSS), version 25 (IBM Corp., Armonk, NY) and to brightstat.com, version 1.3.1 [15]. Descriptive and inferential statistical analyses were done, depending on the variables tested. Significance tests were completed to examine the relationships between variables. To assess the validity of the surgical model, Kendall’s Tau B was computed between the participants’ educational level and the surgical-time measurements.

Three separate repeated measures analyses of variance were conducted to examine the influence of the technique (endoscopic vs. microscopic), task (grasp vs. dissect), training status (consultant, resident or student) and randomization order (endoscope first vs. microscope first) on participants’ surgical performance, attempts and finally damages to the ossicular chain. Time and number of attempts to complete the task as well as damages per task served as dependent variables respectively in the three analyses of variance. Each participant performed the same surgical tasks twice, once endoscopically and once microscopically. *P*-values less than 0.05 were considered to be statistically significant; however, for the purpose of clinical relevance, only effects with observed effect sizes (partial eta-squared, ${\eta}_p^2$) greater than 0.2 are reported.

## Results

### Participants

A total of 33 participants were enrolled in the present study: nine last year medical students, 14 residents and ten ORL-HNS consultants. The consultants had a median surgical experience of 65 (range 4 to > 400) middle ear procedures (microscopic or endoscopic), whereas students and residents were inexperienced with regard to middle ear procedures. The same collective was also assessed regarding anatomical knowledge as previously reported [16]. Independently of the technique and the tasks we observed a statistically significant difference in the time required to complete the tasks between the three levels of training status (*F*_*2, 30*_ = 19.998, *p* < 0.001, ${\eta}_p^2$ = 0.571). The validity of the model was additionally calculated using Kendall’s Tau B. Coefficients ranged from 0.380 to 0.564, with all having *p* < 0.01. The validity of the instrument is supported by these coefficients, especially considering the relatively small sample size.

### Surgical skills assessment

Three separate repeated measurements ANOVAs were conducted with technique and task as within subjects’ factors and training status as well as randomization order as grouping factors. As dependent variables served time and number of attempts to complete the task as well as number of damages made during the task. The dissection tasks took statistically significant less time to be performed compared to the grasping tasks (54.30 s (41.89) versus 79.21 s (63.49), *F*_*1, 27*_ = 37.120, *p* < 0.001, ${\eta}_p^2$ = 0.579). Moreover, the dissection tasks were associated with significantly more accidental damage to the ossicular chain than the grasping tasks (1.05 (1.26) versus 0.30 (0.86), *F*_*1, 27*_ = 19.249, *p* < 0.001, ${\eta}_p^2$ = 0.416).

Time (*F*_*1, 27*_ = 0.276, *p* = 0.604) and attempts (*F*_*1, 27*_ = 1.695, *p* = 0.201) necessary to complete all tasks did not differ statistically significantly between the two techniques (Table 1).

**Table 1:** Surgical time, attempts and damages required for grasping and dissection tasks represented per surgical technique (endoscope vs. microscope) and level of education (students vs. residents vs. consultants). Standard deviation is indicated in brackets

		Grasp		Dissect	
		Endoscope	Microscope	Endoscope	Microscope
Time (sec)	Students	128.2 (73)	149.8 (93.5)	67.4 (45.8)	101.7 (67.3)
	Residents	82.9 (37.7)	59 (29.1)	50.3 (22.7)	60.1 (28.2)
	Consultants	38.6 (20.8)	35.4 (29.8)	25.9 (19.3)	25.8 (15.3)
Attempts	Students	4.1 (1.8)	5.4 (1.7)	3.8 (2.2)	4.1 (1.1)
	Residents	3.6 (1.6)	3.5 (1.4)	2.9 (0.7)	3.7 (1.4)
	Consultants	2.7 (0.7)	2.8 (1.4)	2.6 (1.1)	2.4 (0.7)
Damage	Students	0.44 (0.73)	0.89 (1.96)	1.33 (1.41)	3 (0.87)
	Residents	0.07 (0.27)	0.29 (0.61)	0.71 (0.91)	1.07 (1.14)
	Consultants	0.1 (0.32)	0.2 (0.42)	0.2 (0.42)	0.3 (0.48)

**Fig. 4:**
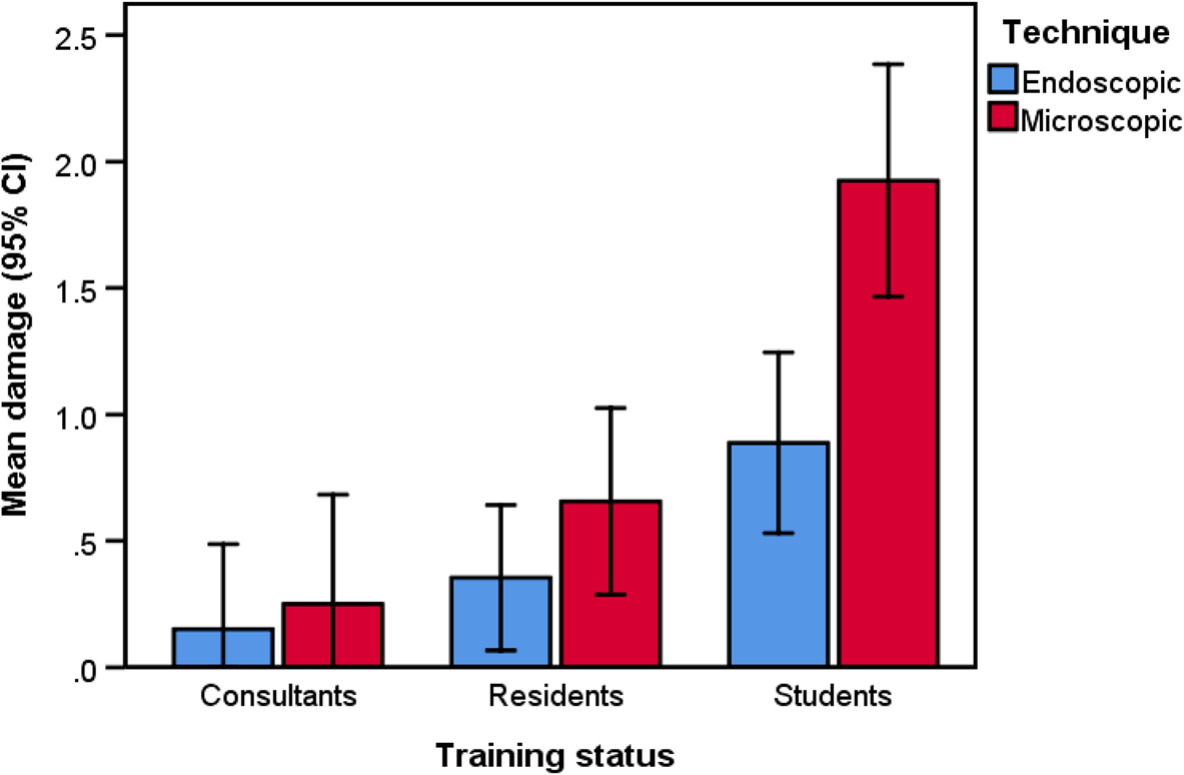
Ossicular chain damages. Accidental damages to the ossicular chain during dissection compared per level of training status and surgical technique. A higher count of damages was observed using the microscopic approach in all educational levels, with a statistically significant difference in the residents/students groups

However, a significant higher number of damages to the ossicular chain was observed with the microscopic as compared to the endoscopic technique (*F*_*1, 27*_ = 14.560, *p* < 0.001, ${\eta}_p^2$ = 0.350). Concerning the damage to the ossicular chain a significant interaction between technique and levels of training status was found (*F*_*1, 27*_ = 4.602, *p* = 0.019, ${\eta}_p^2$ = 0.254). Students (*t*_*7*_ = 2.527, *p* = 0.039, ${\eta}_p^2$ = 0.447) and residents (*t*_*12*_ = 2.369, *p* = 0.035, ${\eta}_p^2$ = 0.319) produced significantly more damage to the ossicular chain with the microscopic technique compared to the endoscopic technique. The comparison among the consultants was not significant (*t*_*8*_ = 1.633, *p* = 0.141, ${\eta}_p^2$ = 0.250). Figure 4 illustrates this interaction.

**Fig. 5:**
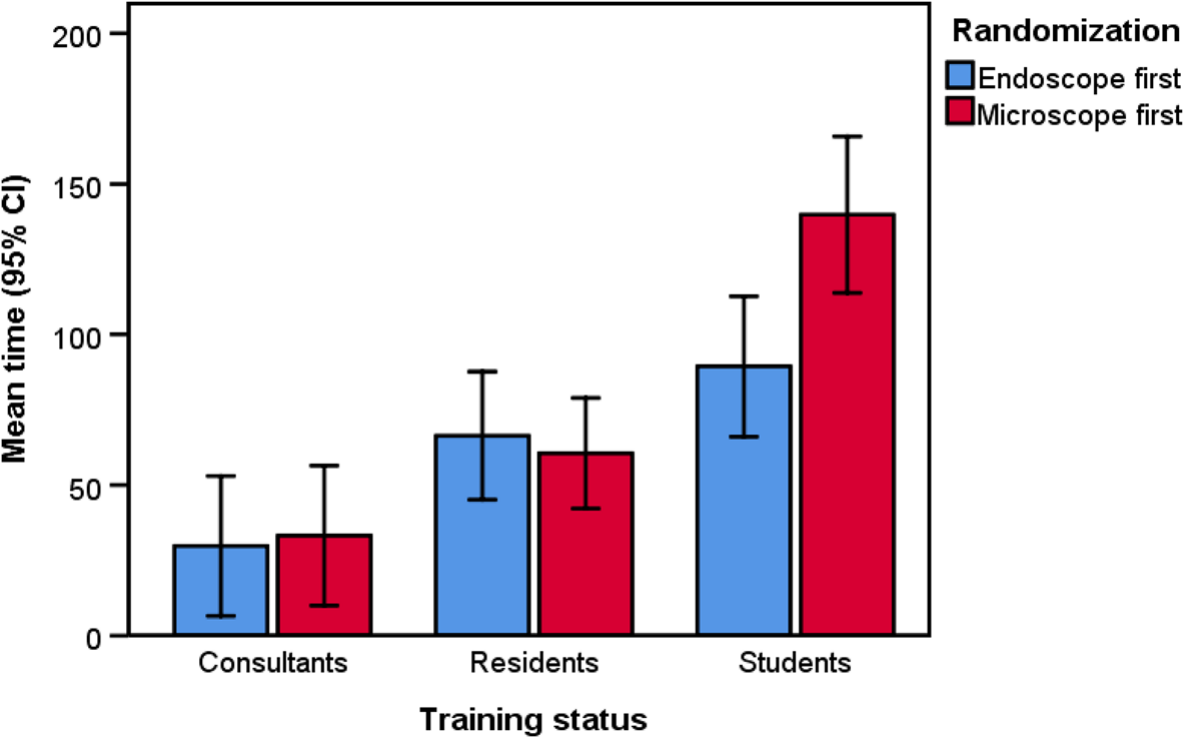
Effect of randomization on surgical time. Comparison of the time required for the surgical tasks, illustrated separately per randomization group (endoscope first versus microscope first) and training status (consultants, residents and students)

Students beginning with the endoscopic technique showed an overall significantly lower amount of time to complete the tasks (*F*_*2, 27*_ = 3.538, *p* = 0.043, ${\eta}_p^2$ = 0.208). Figure 5 depicts this significant interaction between randomization group and level of training status. Moreover, three participants in the microscopic group dislocated one of the plastic ring towards the Eustachian tube orifice during dissection and required repositioning by the tutor. No such interventions were necessary in the endoscopic group.

### Subjective feedback

The means and standard deviations of the answers on the five-point Likert scale to the questions in the feedback survey are summarized in Fig. 3 per education level. In general, we observed a preference of the endoscopic technique mainly in medical students and residents, who were not previously trained in middle ear surgery. This becomes especially evident in Q3 showing an increasing preference for the endoscope with decreasing experience. On the other hand, we observe in Q5 that only well-trained surgeons benefit subjectively from the concomitant use of instrument and suction during the surgical tasks. These questions were part of the same questionnaire investigating the utility of the endoscopic approach regarding teaching middle ear anatomy [16]. However, no previously reported questions are included in this study.

## Discussion

This study compares the endoscopic to the microscopic technique for the acquisition of basic surgical skills in middle ear surgery under controlled conditions regarding given instructions and assessment of surgical performance. The required surgical times to complete each task were highly associated to the educational level (medical student, resident, consultant) and experience of the participants. The used technique had no significant impact on time and attempts required for each task. However, a statistically significant effect was observed regarding accidental damages to the ossicular chain, which was lower in the endoscopic technique. Moreover, medical students performed statistically significantly better when they were first taught the surgical skills using the endoscopic technique. This direct comparison of the endoscopic and the microscopic technique regarding surgical skills acquisition in middle ear surgery has to our best knowledge not been published before. From the results obtained during this standardized investigation several interesting considerations should be discussed.

First of all, the time required to solve the surgical tasks in this temporal bone model were not associated to the technique used. Despite not assessing a full learning curve, the observation of similar operating time at baseline is in our opinion, an argument against the propagated slower learning curve in EES. Moreover, medical students which are completely novice to middle ear surgery show a higher benefit of the endoscopic technique as compared to the microscope regarding surgical skills acquisition. To date, the literature on EES skills acquisition mainly consists on reports by surgeons previously trained in the microscopic technique [17]. However, it would be interesting to investigate the learning curve of young surgeons who make their first experiences in otology using an endoscope in the future. These surgeons do not have a prefixed model of a (microscopic) intervention in their mind and therefore may be more flexible and fast in adopting EES. On the other hand, the hereby investigated model lacks bleeding, which is of course an important issue in the operating room. The one-handed surgical technique in EES requires decent knowledge and skills in managing the bleeding [18, 19], which are different from the microscopic technique and may impede with the learning curve during clinical application. However, it is important to stress, that technical as well as procedural differences should not be confounded with learning curves for young surgeons. Especially, as the subjective perception of a learning curve for a previously not trained surgeon may be very different from an experienced surgeon, who has to learn a new technique.

Second, a statistically significant lower rate of accidental ossicular chain damages in the endoscopic compared to the microscopic technique was observed. The panoramic views provided by the endoscope improves the visibility of the middle ear cleft, which allows to avoid accidental damage to critical structures. Moreover, the learner has the same perspective as the tutor in the endoscopic technique, which allows direct application of the observations and steps taught into his own surgical skills performance. Similarly, the benefits of these wide-angle views in teaching middle ear anatomy were previously reported [16]. Additionally, the lack of depth perception in the endoscopic technique did apparently not interfere with a cautious and precise manipulation of the instruments inside the middle ear. In contrary, the constant visual feedback on the exact position of the ossicles and the instrument allowed the diminution of accidental trauma to the ossicular chain during this assessment. Ultimately, in the endoscopic technique the tutor did not need to intervene for lost dissection rings as compared to the microscopic group, where three salvage interventions were necessary. Due to the overview provided by the endoscope, the participants were able to resolve the problem in case of dislocation themselves.

Lastly, regarding the subjective preference of the participants, a tendency towards the endoscopic technique was observed. Especially residents and medical students preferred the endoscope for educational purposes. In this context, the question arises whether or not the endoscope should already be implied during medical education for instance during anatomical dissections in the first years of the curricula. However, at least during educational courses on middle ear surgery, the use of the endoscopes can be strongly recommended based on our observations.

The main limitation of the present study is the use of a cadaveric model, which of course hinders the complete transfer to the clinical context. However, this approach was necessary to perform this study under standardized and controlled conditions with the aim to eliminate the issues related to surgical skills training in the operating room. Additionally, the used Thiel conservation method [20] offers excellent soft tissue properties, which is particularly suitable for surgical training under live-like conditions [21].

The strengths of this study include the innovative study design addressing for the first time an important issue in postgraduate education of ear surgeons. Moreover, both objective and subjective measures were included and three different levels of expertise assessed using a standardized and established training approach [14].

## Conclusion

The endoscopic approach is useful and beneficial for teaching basic surgical skills, mainly by providing a reduction of damage to surrounding tissues with similar operating times for both techniques. Moreover, medical students performed significantly faster, when first taught in the endoscopic technique. Especially for residents without previous training in ear surgery, the endoscope should be considered to improve surgical skills in the middle ear.

## Data Availability

The dataset supporting the conclusions of this article is included within the article.

## Abbreviations

EES: Endoscopic ear surgery
ORL-HNS: Otorhinolaryngology, Head and Neck Surgery
